# News and Notes

**Published:** 1907-08

**Authors:** 


### NEWS AND NOTES.

Eighteen public bath-houses were formally opened June 24 in Philadelphia.

The Thirty-eighth Annual Session of the Virginia Medical Association will convene at Chase City, Va., November 12, 1907.

The French Congress of Practitioners declared its approval of the plan to charge double rates for professional calls made on Sunday.

The board of trustees of the Grady Hospital has elected J. J. Meador, a prominent business man of Atlanta, to succeed T. F. Brewster as superintendent.

For the six months from January to June, 1907, there have been 1,060,067 death from plague in India alone. This mortality rate is the highest ever recorded.

Seven German Universities admit women on the same terms as men, and at present there are 116 women in the Medical departments. This is an increase of 56 over the preceding year.

Dr. J. P. Sutherland, Dean of the Boston University Medical School, has announced that beginning next year the course for the degree M. D. will be increased from four to five years.

In response to the Petit Parisien as to who was the greatest Frenchman of the 19th Century, 15,000,000 votes were received. Pasteur came in an easy first, with a million more votes than Victor Hugo.

The Atlanta School of Medicine is installing an epidiascope for illustrating lectures, etc. It is said to be the most perfect instrument of the kind known, there being only five or six in America.

Mrs. Bertha Rayner Frank has endowed a medical scholarship at the University of Maryland, in memory of her late husband, to be known as "the Dr. S. Leon Frank Scholarship." The interest of $2,500 is to be applied annually to this scholarship.

---

The death of Professor Max Schuller, of the University of Berlin, occurred on June 18, 1907, in the sixty-sixth year of his age. Professor Schuller has devoted several years to investigations in the aetiology of cancer in the effort to isolate the cancer bacillus.

The Southern Railway Surgeon's Association met in Washington, D. C., May 30th, and elected the following officers: Pres., Dr. H. T. A. Lemon, Washington; Vice-Prest., Dr. C. H. Starkel, and Dr. T. J. Hoppel; Sec. and Treas., Dr. J. U. Ray, re-elected. The next meeting will be held in Birmingham, Ala.

---

The Texas State Medical Association has elected the following officers for the coming year: President, Dr. C. E. Cantrell, Greenville; Vice-Presidents, Dr. H. S. Barnes, Tulia; Dr. D. S. Weir, Beaumont, and Dr. A. B. Small, Waxahachie; Treasurer, Dr. C. A. Smith, Texarkana; Secretary (for three years), Dr. I. C. Chase, Fort Worth.

---

The Editor of the St. Louis Medical Review has announced that with the issue of July the 6th, the weekly publication of this Journal will cease and that henceforth it will continue as a monthly. We deeply regret the necessity for this change of an independent weekly journal which has been so ably edited and wish their publication success as a monthly magazine.

---

The City Council of Atlanta has elected the following city physicians: First Ward, Dr. B. F. Harwell; Second Ward, Dr. E. van Goidtsnoven; Third Ward, Dr. M. C. Martin; Fourth Ward, Dr. I. T. Catron; Fifth Ward, Dr. L. H. Jones; iSxth Ward, Dr. J. G. Wilkins; Seventh Ward, Dr. A. H. Lindorme; Eighth Ward, Dr. J. R. Garner.

The Texas State Board of Health has determined to deny admission to that State to all persons in an advanced stage of tuberculosis. This is not intended to debar those who are slightly affected and have a chance to recover in the arid region of west Texas. They simply wish to exclude those so far advanced that they are a menace to persons around them and have little chance of recovery.

---

Mrs. Russell Sage has given $300,000 to found the Russell Sage Institute of Pathology to perform the duties of pathologists for the City Hospital and the City Home for the department of charities on Blackwell's Island. Mrs. Sage makes a special request that this institution make a specialty of research into the problems of all diseases, and more especially to those incident to old age.

---

The next annual course of lectures on the foundation established by Doctor and Mrs. C. A. Herter, of New York, before the medical department of the Johns Hopkins University, will be given by Dr. Edward A. Schafer, professor of physiology in the University of Edinburgh, Scotland, who was for 16 years professor of physiology in University College, London, and general secretary of the British Association from 1895 to 1900.

---

The use of basement rooms for school purposes in Atlanta led the Fulton County Medical Society to appoint a committee consisting of Drs. Dunbar Roy, J. C. Olmstead, Wesley Taylor, and Theo. Toepel, to investigate and report on the conditions existing. The committee visited all the public schools in the city and found that the rooms were damp, poorly lighted and adjoining lavatories. This protest should lead to improvements and increased facilities before the fall term of school opens.

---

The Interstate Medical Journal (St. Louis) announces the purchase of the St. Louis Courier of Medicine, one of the oldest medical journals in the West, and its consolidation with the Interstate on July 1st.

The St. Louis Courier of Medicine was established in 1879 by an association of prominent St. Louis physicians. It has always commanded a large following throughout the West and South, and held the respect and esteem of the entire profession of this country.

